# Whole genome DNA methylation: beyond genes silencing

**DOI:** 10.18632/oncotarget.13562

**Published:** 2016-11-24

**Authors:** Roberto Tirado-Magallanes, Khadija Rebbani, Ricky Lim, Sriharsa Pradhan, Touati Benoukraf

**Affiliations:** ^1^ Cancer Science Institute of Singapore, National University of Singapore, 117599 Singapore, Singapore; ^2^ Computational Systems Biology Team, Institut de Biologie de l’Ecole Normale Supérieure (IBENS), INSERM, Ecole Normale Supérieure, PSL Research University, 75005 Paris, France; ^3^ New England Biolab Inc., Ipswich, MA 01938, USA

**Keywords:** DNA methylation, bisulfite sequencing, gene regulation, chromatin modeling, imprinting

## Abstract

The combination of DNA bisulfite treatment with high-throughput sequencing technologies has enabled investigation of genome-wide DNA methylation at near base pair level resolution, far beyond that of the kilobase-long canonical CpG islands that initially revealed the biological relevance of this covalent DNA modification. The latest high-resolution studies have revealed a role for very punctual DNA methylation in chromatin plasticity, gene regulation and splicing. Here, we aim to outline the major biological consequences of DNA methylation recently discovered. We also discuss the necessity of tuning DNA methylation resolution into an adequate scale to ease the integration of the methylome information with other chromatin features and transcription events such as gene expression, nucleosome positioning, transcription factors binding dynamic, gene splicing and genomic imprinting. Finally, our review sheds light on DNA methylation heterogeneity in cell population and the different approaches used for its assessment, including the contribution of single cell DNA analysis technology.

## INTRODUCTION

**Figure 1:**
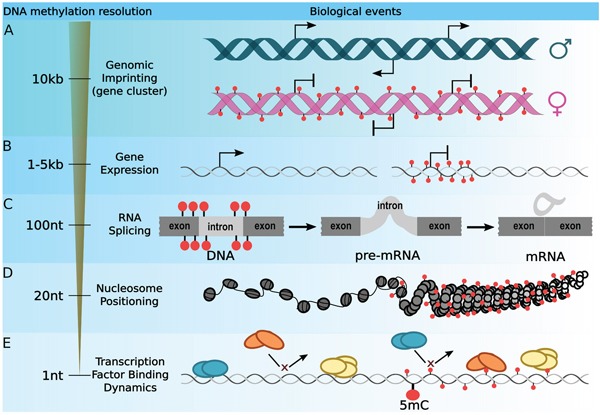
Optimizing the DNA methylation resolution according to a biological context The most widely used strategy for integrating DNA methylation with gene regulation is to average CpG methylation signal throughout wide loci. **A**. The study of the imprinting regions can be achieved by averaging DNA methylation signal of loci ranging from 1 kb to 10 kb. **B**. Interplay between gene expression and DNA methylation are usually drawn by studying the DNA methylation level within the 1 kb to 5kb region surrounding the TSS. **C**. However, DNA methylation is involved in many other mechanisms. Extending the DNA methylation resolution to a 100 bp around splicing sites enables the investigation of exon inclusion. **D**. On the other hand, a 20 bp resolution was established to be optimal for studying the interplay between DNA methylation and nucleosome positioning. **E**. Finally, DNA methylation plays a key role in the recruitment of transcription factor and it has been shown that methylation of a single cytosine can affect protein/DNA binding affinity.

DNA methylation is a heritable covalent chemical modification of DNA, crucial for numerous biological processes such as gene regulation, cell fate decisions and disease development [1]. Of particular note is the use of DNA methylation inhibitors as powerful therapeutic agents in the treatment of myelodysplastic syndrome and, with lesser success, of solid tumors [2, 3]. The detailed molecular mechanisms underlying the effects of DNA methylation on chromatin folding and accessibility as well as on gene regulation remain poorly understood. The dogma portraying the function of DNA methylation as an inhibitor of gene expression is still pervasive. Global and unbiased DNA methylation analysis protocols such as whole genome bisulfite sequencing, empowered by the advent of high-throughput sequencing, revealed a more sophisticated role of DNA methylation, with gene silencing representing only one facet of its consequences. Increasing evidence suggests that DNA methylation is not only associated with gene repression, but also with gene activation [4], splicing regulation [5], nucleosomes positioning [6–8], and the recruitment of transcription factors [9–12]. Together, this multiplicity of functions suggests that DNA methylation is more accurately described as a process akin to a cellular epigenetic memory [13–16]. DNA methylation is widely analyzed in the CpG context, due to the fact that 80 % of methylation events occur at CpG sites in mammalian genomes. However, in plants, only 24 % of CpG sites are methylated, while 6.7 % and 1.7 % of CHG and CHH (where H = A, T or G) are methylated, respectively [17]. The symmetry properties of CpG and CHG motifs imply a double-stranded DNA methylation, whereas methylation of the asymmetric CHH motif refers to single-stranded DNA methylation. The functions of CHG and CHH methylation are still unclear. Recent studies revealed that in *Arabidopsis*, CHH methylation occurs predominantly at the transposable elements, and has been involved in epigenetic inheritance [18], as well as in the prevention of transposon jumping during development [19]. In mammals, non-CpG methylation is detected at perceptible levels only in a few cell types, including neuronal cells during brain development and after neurogenesis [20]; in embryonic stem cells and induced pluripotent stem cells [21]. Non-CpG methylation may thus also play a role in X-chromosome inactivation. Furthermore, the striking correlation between methylation patterns in CHG and CHH contexts and human cells suggests that methylation in these contexts might be maintained by a common machinery, in contrast to plants [21]. Furthermore, asymmetric DNA methylation is apparently enriched in the transposable elements SINEs and LINEs, but not for LTRs. So far, because of technological limitations and of the early dogma limiting DNA methylation to its sole gene silencing function, numerous studies have mainly focused on the promoter regions (particularly CpG island promoters). These biases are still evident in current microarray designs aiming at deciphering the state of genome-wide DNA methylation. When correlating DNA methylation status and gene transcription levels, CpG methylation scores are usually averaged throughout the promoters or CpG islands, resulting in a robust estimation of differential DNA methylation call on large regions at the expense of diluting the methylation signal on local loci. Sequencing-based technologies resulted in a dramatic increase in resolution from CpG islands (CGI) level down to the level of single cytosine in the CpG, CHG or CHH contexts, shedding a new light on the various biological functions of DNA methylation. These discoveries were made possible by the development of computational strategies not restricted to promoter methylation/gene expression associations [22]. Methodologies used in the recent studies have revealed the necessity of tuning DNA methylation resolution into an adequate scale to ease the integration of DNA methylation information with other chromatin features. This review covers current major DNA methylation analytical schemes. In particular, we discuss optimal resolution choices associated with the study of each aspect of DNA methylation-related biological processes (Figure 1).

### DNA methylation and gene expression

Early perception on the function of DNA methylation has been linked to gene expression [23, 24]. In the 80's, it was reported that promoters hypermethylation correlates with decreased expression levels of downstream genes, such as γ-globin locus [25]. Most of the studies used Southern blot techniques then, which allowed measurement of DNA methylation at the resolution of about 1 kb. These promoter-centric studies substantiated the dogma associating DNA methylation with gene repression. Therefore, as approximately 70 % of annotated promoters overlap a CGI [26], biotechnology companies have developed microarrays containing probes that target preferentially gene promoters [27]. Recent genome-wide studies have demonstrated the soundness of the dogma and succeeded in characterizing genes directly inactivated by DNA methylation [28, 29]. Some studies focus mainly on the gene promoter analysis using various resolution levels. For instance, using the reduced representation of bisulfite sequencing (RRBS) method, which allows interrogation of about 30 % of CpG sites that overlap 65 % of human genome promoters [30], Amabile *et al*. have identified around 500 genes involved in chronic myeloid leukemia (CML) progression by analyzing DNA methylation on promoters [31]. This CML epigenetic signature is characterized by averaging methylation levels of CpG sites located in regions ranging from -1.5 kb to +0.5 kb (i.e. 2 kb-window) around the transcription start sites (TSS). This example shows how broad DNA methylation can change, particularly within the gene promoter regions, which determine the phenotype. With another strategy, Hodges *et al*. [28] studied DNA methylation dynamics during the hematopoietic development using the whole genome bisulfite sequencing method (WGBS) [32]. A 100 bp sliding window was applied to average out CpG methylation levels, which allows comparison of DNA methylation patterns across all TSS regions, regardless of the variations in CpG sites distributions throughout those loci. This strategy permitted the characterization of the typical TSS region (±4 kb) methylation pattern. They reported that DNA methylation level within the ±1 kb region surrounding the TSS showed the greatest correlation with gene repression. Notably, an analysis using GBSA (Genome Bisulfite Sequencing Analyser) [33] substantiated this observation. Interestingly, DNA methylation/gene repression correlation was evident at 1 kb downstream of the TSS of the genes. This observation corroborates another study where DNA methylation of the first exon is shown to be associated with transcriptional gene repression [29]. Refining DNA methylation analysis to 100 bp resolution demonstrated that DNA methylation patterns surrounding TSS are not homogeneous, suggesting that once methylated, some DNA regions, including the first exon, are subjected to gene repression more than the others, and might play a specific role in gene inactivation. In fact, the first exon-intron region of active tissue-specific genes was found to be enriched in the di-methylation of lysine 4 at histone 3 (H3K4me2), which is a predominant signature of gene regulatory elements [34].

Paradoxically, DNA methylation is also associated with gene activation, when it occurs within the transcribed regions [35]. Apparently, gene body methylation enhances transcription. Similarly, in an *in vitro*-induced differentiation study of human embryonic stem cells, a large group of 3′ CGI that underwent an increase in DNA methylation actually correlated with increased expression of these genes [36]. These relationships exhibit the multi-faceted and complexity of DNA methylation roles in gene regulation and the importance of the genome structure integration.

### DNA methylation dictates nucleosome positioning

An obvious mechanism in which DNA methylation participates in gene regulation is by nucleosome positioning, i.e. restricting the accessibility for protein complexes to DNA regulatory regions (such as gene promoters or TSS). An early depiction of the involvement of DNA methylation in nucleosome positioning was described in a study whereby DNA methylation in a 3x(CpG) element was targeted, leading to the depletion of a neighboring nucleosome [37]. Although it has been reported that methylation of DNA increases its stiffness which might alter nucleosomal formation [8, 38, 39], numerous studies based on whole-genome approaches have exhibited enrichment in methylated cytosines on the histone-bound DNA sequence. In one report, a 10 bp interval of methylated and unmethylated CpGs in the nucleosomal DNA was observed [6], showing that unmethylated CpG dinucleotides occur principally in the minor grooves facing away from the histone octamer, whereas the methylated counterpart is mostly seen in the minor groove in proximity to the octamer complex promoting nucleosome stability [6, 40]. A recent analysis has demonstrated that methylation of CpGs in the major groove of the DNA wrapped around the histone octamer greatly influences the nucleosome dynamics towards a more open structure, while the methylation state of CpGs located in the minor grooves has a negligible effect [8]. Interestingly, the nucleosome occupancy within exons correlates with the local CG density [41].

To date, NOMe-Seq is the most robust method to study the relationship between nucleosome positions and DNA methylation status simultaneously in a genome [7]. This sequencing-based method was used to investigate nucleosome structure and DNA methylation at CGIs in oncogenes, by combining the usage of a GpC methyltransferase (M.CviPl) to obtain nucleosome positioning information based on enzyme accessibility to GpC sites, and bisulfite DNA treatment to determine the methylation status of cytosines [42]. A prominent anti-correlation between DNA methylation and nucleosome occupancy at CTCF binding regions was revealed in different cell lines by averaging NOMe-Seq signal using a 20 bp sliding window. However, this anti-correlation was not observed at gene promoters. It was reported that the 20 bp resolution is optimum as this is the average distance between two adjacent CpG dinucleotides. In subsequent studies, by using NOMe-Seq and examining the profiles with 100 bp windows at 20 bp spacing, the interplay of DNA methylation and nucleosome occupancy at the enhancer regions of cancer cell lines was determined [43, 44]. An additional study observed the aggregate profile of 5mC coming from bisulfite-sequencing around CTCF binding sites as well as their MNase-Seq profiles on different cell lines using a single base pair resolution (1 bp) [45]. Their results were strikingly similar to the ones reported in the initial NOMe-Seq study, further supporting that a 20 bp resolution is adequate to resolve the nucleosome positioning/DNA methylation interplay and that using a narrower resolution will yield similar results.

### DNA methylation and transcription factor binding dynamics

Transcription factors (TFs) are key effectors in the activation of transcription. Their functions rely on binding to the DNA upon recognition of a particular nucleotide motif through steric interactions between the TF protein domains and the DNA molecule. It has been long known that chemical modifications to the DNA bases can either increase or restrain these interactions [46]. DNA methylation in the vicinity of TSS region is a common proxy for the transcriptional status of a gene. It has been reported that the methylation status of only 16 % of CpG sites surrounding the TSS correlates negatively with the expression of their corresponding genes [47]. Moreover, these CpG sites are generally avoided at predicted TF binding sites, especially at the binding sites of known TF with a repressive function. It is often assumed that the reason behind this negative correlation is the difficulty of TFs in binding to methylated cytosines. For instance, the abrogation of DNA methylation in murine stem cells by knocking out DNA methyltransferases DNMT1, DNMT3A and DNMT3B increases the amount of NRF1 binding events dramatically [48]. However, the relationship between DNA methylation and TF binding is complex and is often dependent on cell signaling and post-translational modifications of TF. By investigating the mechanism of interaction using a high-throughput array-based technology, Hu *et al*. evaluated the effect of single cytosine methylation on 154 TF DNA binding motifs containing at least one CpG site [11]. This study revealed that, depending on the TF, DNA methylation could either hamper or enhance TF/DNA interactions. Furthermore, some TFs showed the ability to bind different motifs depending on their methylation status. Likewise, previous studies have reported that DNA methylation in the close vicinity of TF binding sites, which do not contain CpG site, might also alter the strength of TF/DNA interaction. This is the case for AP-1, a TF complex composed of cFOS and cJUN that recognizes the TGANTCA motif. In fact, this complex appears to lose its ability to bind DNA when a CpG site adjacent to the core-binding motif is methylated [10]. Similarly, although methylation within the consensus Sp1-binding site does affect Sp1/Sp3 binding, methylation adjacent to the core Sp1 motif induces a significant decrease in Sp1/Sp3 binding [9]. Interestingly, this phenomenon also occurs in an allele-specific manner. For instance, YY1 binding events are modulated by DNA methylation in a parent-of-origin-specific fashion, in such a way that only CpGs close to the binding site of the maternal allele are methylated, preventing YY1 to bind only the maternal allele [49].

Furthermore, it is worth mentioning that enzymatic modification of cytosine is a complex dynamic involving DNMT1, DNMT3A and DNMT3B methyltrasferases, which methylates cytosines (5mC), and the TET family of cytosine oxygenase enzymes, which oxidizes 5mC to 5-hydroxymethylcytosine (5hmC), subsequently to 5-formylcytosine (5fC) and finally to 5-carboxycytosine (5caC) [50, 51]. These oxidized derivatives might also hinder TF binding. In principle, the presence of these derivatives can alter the way in which proteins bind to their recognition sequences in DNA by strengthening the interactions, weakening them, or by abolishing them completely. For example, Klf4 shows the strongest binding to fully methylated DNA, with slightly higher affinity (approximately 1.5-fold) than that of the unmodified DNA, and in each oxidation event, from 5mC to 5hmC to 5fC to 5caC, resulting in progressively weaker binding (by factors of ~2, 3, and 6, respectively) [52].

The function of these oxidized derivatives of 5mC is still under discussion and many consider these changes as a transitory process leading to DNA demethylation [53]. However, recent observations revealed that 5hmC is enriched in the short interspersed elements (SINEs) and long terminal repeat (LTR) regions, while 5fC and 5caC seemed to be more prevalent within the satellite repeats regions of the genome [54]; and that the DNA binding affinity of numerous proteins increases in the presence of these oxidized derivatives of 5mC [55, 56], suggesting that 5hmC, 5fC and 5caC possess functional epigenetic roles. Nonetheless, a significant drawback of WGBS is the inability to distinguish 5mC from 5hmC, because sodium bisulfite treatment is unable to convert both methylated states to uracil [57]. This frequently neglected limitation should be taken into account when interpreting bisulfite-converted data.

### DNA methylation and gene splicing

The majority of eukaryotic genes give rise to several isoforms. Aberrant splicing can lead to extreme phenotypes such as spinal muscular atrophy [58], suggesting a tight regulation during exons selection. Studies of the splicing process began about 40 years ago, although many of the mechanisms and signals controlling are still being actively investigated. It has been shown recently that gene body DNA methylation controls gene transcription and exon splicing [59]. Using methylation array technologies, a study showed that tissue-specific differentially methylated regions in mouse are preferentially located in exons and introns of protein coding genes with known alternative splicing variants [60]. Many of the splicing events in the human genome occur co-transcriptionally while the precursor mRNA remains associated with the chromatin until the introns are excised [61, 62]. This is related to findings suggesting that chromatin marks and structures provide the signals for exons selection [63]. Remarkably, genes with similar GC content within exons and introns exhibit significant decrease in CpG methylation levels of the 100 bp region towards the introns from the exon-intron junctions, compared to the rest of the intron; and a striking enrichment of DNA methylation levels in the 20 bp region surrounding the 5′ and 3′ splice sites [64].

Recent findings highlighted the essentiality of DNA methylation status in the recruitment of protein factors responsible for splicing signals such as CTCF and MeCP2 [59]. Depending on the context, DNA hypermethylation prevents CTCF from binding to the transcribed region, accelerating the processivity of Pol II which is translated into a higher frequency of exon exclusion [5]. In contrast, CpG methylation increases MeCP2 binding affinity to DNA, leading to the recruitment of histone deacetylases, decreasing Pol II activity and enhancing exon inclusion successively [65].

### DNA methylation regulates genomic imprinting

Imprinting refers to the targeted inactivation of a genomic region in one of the parental chromosomes. The implications of imprinting in intergenerational inheritance are currently under intense investigation due to its medical implications, such as metabolic changes as a consequence of the diet of an ancestor. There is also emerging evidence showing DNA methylation as one of the major players in this biological process [66]. DNA methylation can establish differential parental methylation during gametogenesis *via de novo* methyltransferase activity (DNMT3A/B) in the testes or ovaries. DNA methylation can be inherited to the next generation by maintaining the methylation status with the help of DNMT1 after gametogenesis and also during subsequent embryonic cell divisions. Demethylation can occur passively after DNA replication if the mark is not maintained. Nonetheless, the active process of demethylation remains largely unveiled and is still being investigated.

Imprinted genes are generally clustered in DNA regions ranging from 100 kb to 3700 kb [67]. These imprinted loci can contain 3 to 12 genes. Strategies which consist of scanning methylC-seq datasets with a resolution of 5 kb have succeeded in identifying known and several novel imprinting regions [68]. Most of the allele-specific DNA methylation (ASM) identification methods segregate alleles by genotypic variations (e.g. SNP). However, not all imprinting regions contain such variations. To overcome this limitation, Fang *et al*. have recently developed a statistical tool to identify ASM from bisulfite sequencing dataset based on the assumption that the methylation signal derived from sequenced reads can be de-convoluted into two distinct patterns [69]. In sequential order, this tool fits signal from the bisulfite sequencing data with a single-allele model, followed by an allele-specific model, and determines which model provides the best fit to the data using the Bayesian Information Criteria (BIC) method. Furthermore, they implemented an Expectation Maximum (EM) algorithm to infer the probabilities of the allele of origin for each methylation read. Using a resolution of 10 CpGs and 20 bp, this strategy allows identification of ASM from a wide range of human cell types. Although differentially methylated alleles in SNP regions in the genome have been effectively used to infer ASM, this strategy is based on the assumption that DNA methylation patterns remain consistent during cell division, and therefore is limited to the analysis of homogeneous cell populations.

### DNA methylation heterogeneity patterns in cell population

Managing cellular heterogeneity information, particularly within tumor samples, represents one of the greatest challenges in (epi)genomic analysis. Promising statistical algorithms that can identify DMRs from individual tumor methylome samples without genomic variation information or prior knowledge from other datasets are becoming crucial tools for the efficient analysis of DNA methylation signal derived from heterogeneous cell populations. Software packages such as MethylPurify [70] allow segregation of DNA methylation patterns using complex deconvolution analysis in regions with bisulfite reads showing discordant methylation levels. Similarly, DMEAS [71, 72] uses a Shannon entropy model to assess the DNA methylation heterogeneity within DNA loci consisting of at least 4 CpG sites sequenced within the same reads. These methods require a DNA methylation signal at the nucleotide resolution and allow inferring of cell heterogeneity levels (i.e. tumor purity from tumor samples alone), which permit better characterization of DMR across samples. However, the increasing length of reads generated by the latest sequencers (> 20 kb) will allow the combination of DNA methylation patterns with SNP information and improve single molecule-level detection as well as phasing of DNA methylation.

### Towards single cell methylome analysis

Methylome analysis of heterogeneous cell populations such as tumor samples, which are known to bear many different cell subtypes; or during developmental and differentiation processes, where cells are unlikely to differentiate in a synchronous manner, remains challenging due to the difficulty in deconvoluting DNA methylation signals of cell sub-populations. A strategy to overcome this obstacle is to measure the methylome at the single cell level [73]. However, bisulfite treatment degrades a non-negligible portion of DNA, and this situation worsens in single cell experiments due to the small quantity of DNA starting material. Indeed, bisulfite treatment in single cell protocols results in the loss of vast portions of the genome, which is translated into poor mapping efficiencies (of about 20 % or total library size) covering between 18 % and 50 % of all the CpGs in the human genome [74]. Nonetheless, despite a reduced mapping efficiency, a striking correlation was observed when looking at the methylation levels between single cell samples and population samples at 2-kb windows (average R = 0.95), suggesting that DNA methylation levels are consistent between different samples. Although single cell methylome analysis protocols have to be optimized, this method has already revealed interesting novel epigenetic mechanisms. For instance, by combining single cell genotyping, gene expression and DNA methylation profiles in a single assay, a research group was able to identify the DNA methylation changes driven by mutations in the EGFR gene from primary tumor samples that would have been otherwise masked by the heterogeneity of the tissue [75].

### Conclusion and perspectives

In recent years, whole genome methylation assays have gained recognition in clinical and biomedical research. Working with DNA has more advantages over RNA assays, not only due to the importance of DNA methylation in genome regulation, but also because the former is more stable, thereby simplifying sample collection, processing, transport, and storage. A second advantage is that when performing assays in heterogeneous cell populations, the amount of DNA extracted is proportional to the cell count. On the contrary, for RNA, a small subpopulation of cells with high transcription rates can mask the profiles of the others. Such is the case of transcriptome assays on tumors infiltrated by immune cells, where the RNA profiles arise mostly from T-cells or macrophages rather than neoplastic cells. However, the spectrum of the roles of DNA methylation in biological processes is far from fully drawn. Indeed, as opposed to previous conceptions, DNA methylation is not a “molecular lock” that prevents gene expression, but rather a complex feature that affects many genomic features dynamically [13, 15, 16]. A myriad of sequencing-based approaches to investigate the methylation status of the cells have been developed [76], but the bottle-neck remains in the downstream analysis and data interpretation. One of the major limitations lies in the sample preparation and the use of bisulfite reagent for deamination. Although sodium bisulfite-based sequencing accurately determines DNA methylation, it cannot distinguish between 5mC and 5hmC. Furthermore, degraded DNA and deaminated adducts are poor products for library construction and hence are not ideal for accurate quantification, particularly for single cell experiments. However, technologies for single molecule DNA sequencing for mammalian epigenome analysis are being developed.

During the recent years, DNA methylation in the CpG context has been shown to act on numerous biological processes, including tumorigenesis, depending on the 5mC density and genomic location. Nonetheless, various investigations have to be performed to explore the role of CHG and CHH methylation in mammalian cells.

To date, the main leverage to maximize biological knowledge extraction from methylome datasets derived from bisulfite sequencing methods is the optimization of DNA methylation resolution measurement according to a specific biological process of interest.
